# Calculating sample entropy from isometric torque signals: methodological considerations and recommendations

**DOI:** 10.3389/fphys.2023.1173702

**Published:** 2023-06-01

**Authors:** Peter C. Raffalt, Jennifer M. Yentes, Sandro R. Freitas, João R. Vaz

**Affiliations:** ^1^ Department of Biology, University of Southern Denmark, Odense, Denmark; ^2^ Department of Nutrition, Exercise and Sports, University of Copenhagen, Copenhagen, Denmark; ^3^ Department of Kinesiology & Sport Management, Texas A&M University, College Station, TX, United States; ^4^ Faculdade de Motricidade Humana, Universidade de Lisboa, Lisboa, Portugal; ^5^ Egas Moniz Center for Interdisciplinary Research (CiiEM), Egas Moniz School of Health & Science, Caparica, Almada, Portugal

**Keywords:** regularity, time series, nonlinear analysis, motor control, muscle contraction

## Abstract

We investigated the effect of different sampling frequencies, input parameters and observation times for sample entropy (SaEn) calculated on torque data recorded from a submaximal isometric contraction. Forty-six participants performed sustained isometric knee flexion at 20% of their maximal contraction level and torque data was sampled at 1,000 Hz for 180 s. Power spectral analysis was used to determine the appropriate sampling frequency. The time series were downsampled to 750, 500, 250, 100, 50, and 25 Hz to investigate the effect of different sampling frequency. Relative parameter consistency was investigated using combinations of vector lengths of two and three and tolerance limits of 0.1, 0.15, 0.2, 0.25, 0.3, 0.35, and 0.4, and data lengths between 500 and 18,000 data points. The effect of different observations times was evaluated using Bland-Altman plot for observations times between 5 and 90 s. SaEn increased at sampling frequencies below 100 Hz and was unaltered above 250 Hz. In agreement with the power spectral analysis, this advocates for a sampling frequency between 100 and 250 Hz. Relative consistency was observed across the tested parameters and at least 30 s of observation time was required for a valid calculation of SaEn from torque data.

## Introduction

Measurement of isometric force or joint torque is a commonly used method for investigation of human motor control. Traditionally, quantification of spatial characteristics of the produced force using linear measures such as mean, range, standard deviation and coefficient of variation has been used to elucidate motor control decrement following various pathological conditions or aging (Vaillancourt et al., 2002; Tracy et al., 2005; Vieluf et al., 2013). However, these measures ignore the force fluctuations over time during a continuous isometric contraction (Slifkin and Newell, 1999; Stergiou, 2004). Within the last two decades, there has been an increasing interest for the nature of these fluctuations, as they have been theoretically linked to a deeper insight of the underlying motor control (Lipsitz and Goldberger, 1992; Newell and Corcos, 1993; Stergiou and Decker, 2011). Specifically, quantification of the regularity and complexity of isometric force using entropy measures has revealed differences in the motor control of various age groups (Slifkin and Newell, 1999; Deutsch and Newell, 2001; 2003; Challis, 2006).

While the implementation of entropy measures such as sample entropy (SaEn) is relatively straight forward, there are several methodological choices which can bias the outcome and potentially invalidate the results interpretation (Yentes and Raffalt, 2021).

SaEn requires the selection of three input parameters: the length *N* of the investigated data series, the vector length *m* used for comparisons across the time series, and the tolerance radius *r* for determination of similar vectors (Richman and Moorman, 2000; Yentes and Raffalt, 2021). Additionally, the recording of biological signals includes a choice of the appropriate sampling frequency while keeping the Nyquist theorem in mind (Hamill et al., 1997).

Substantially different methodological choices have been reported in biomechanical and physiological literature when applying entropy measures on isometric force recordings. This includes sampling frequencies between 30 and 1,000 Hz, *m* of 2, *r* of 0.1–0.2 times the standard deviation, and *N* of 220–5,000 data points (Slifkin and Newell, 1999; Vaillancourt and Newell, 2003; Challis, 2006; Rose et al., 2013; Pethick et al., 2015; Oliveira et al., 2022; Bauer et al., 2023). These differences in methodology potentially invalidate comparison of results and conclusion across studies (Yentes and Raffalt, 2021). Previously, we have investigated the consequences of different methodological choices in kinematics signals obtained from walking and observed that both the sampling frequency and input parameters were crucial for the analysis output (Yentes et al., 2013; 2018; McCamley et al., 2018; Raffalt et al., 2019). Recent discussions in the literature have also emphasized the importance of the awareness to these methodological choices (Hunter et al., 2021; Yentes et al., 2021).

To capture the temporal evolution of any given biological phenomenon requires sufficient observation time for the dynamics of the phenomenon to unfold. This is not to be confused with data length. While data length is the number of data points recorded, observation time is the duration (i.e., in seconds or minutes) the dynamics of the phenomenon is captured. For continuous signals, data length can be increased by increasing the sampling frequency without increasing the observation time. However, if the observation time is insufficient to capture the dynamics of the phenomenon, increasing the sampling frequency will not compensate for this.

To ensure comparability and validity of future studies using SaEn on isometric force or torque signals, methodological guidelines are needed. These guidelines should address the following topics: 1) selection of the appropriate sampling frequency based on a power spectral analysis to establish which frequencies contain the majority of the information in the signal and on the operating time scale of the biological phenomenon in question, 2) selection of appropriate input parameters (*m*, *r* and *N*) to ensure relative parameter consistency, such that changes in input parameters do not lead to change in the between-group or between-test condition relationship of the entropy outcomes, and 3) selection of the appropriate observation time to capture sufficient information about the dynamics of the biological phenomenon. Therefore, the purpose of the present study was to determine the appropriate methodological approach of the use of SaEn on isometric torque signals. This was achieved by investigating 1) the effect of different sampling frequencies, 2) the effect of different input parameters, and 3) the effect of different observation times.

## Materials and methods

### Subjects

Fifty male professional football (soccer) players (22.3 ± 5.3 years, 1.82 ± 0.08 m, body mass: 74.7 ± 9.0 kg) were recruited from a convenience sample. Injured or in-recovery athletes did not participate in the present study. This study was approved by the local ethics committee (21/2016).

### Experimental setup

**FIGURE 1:**
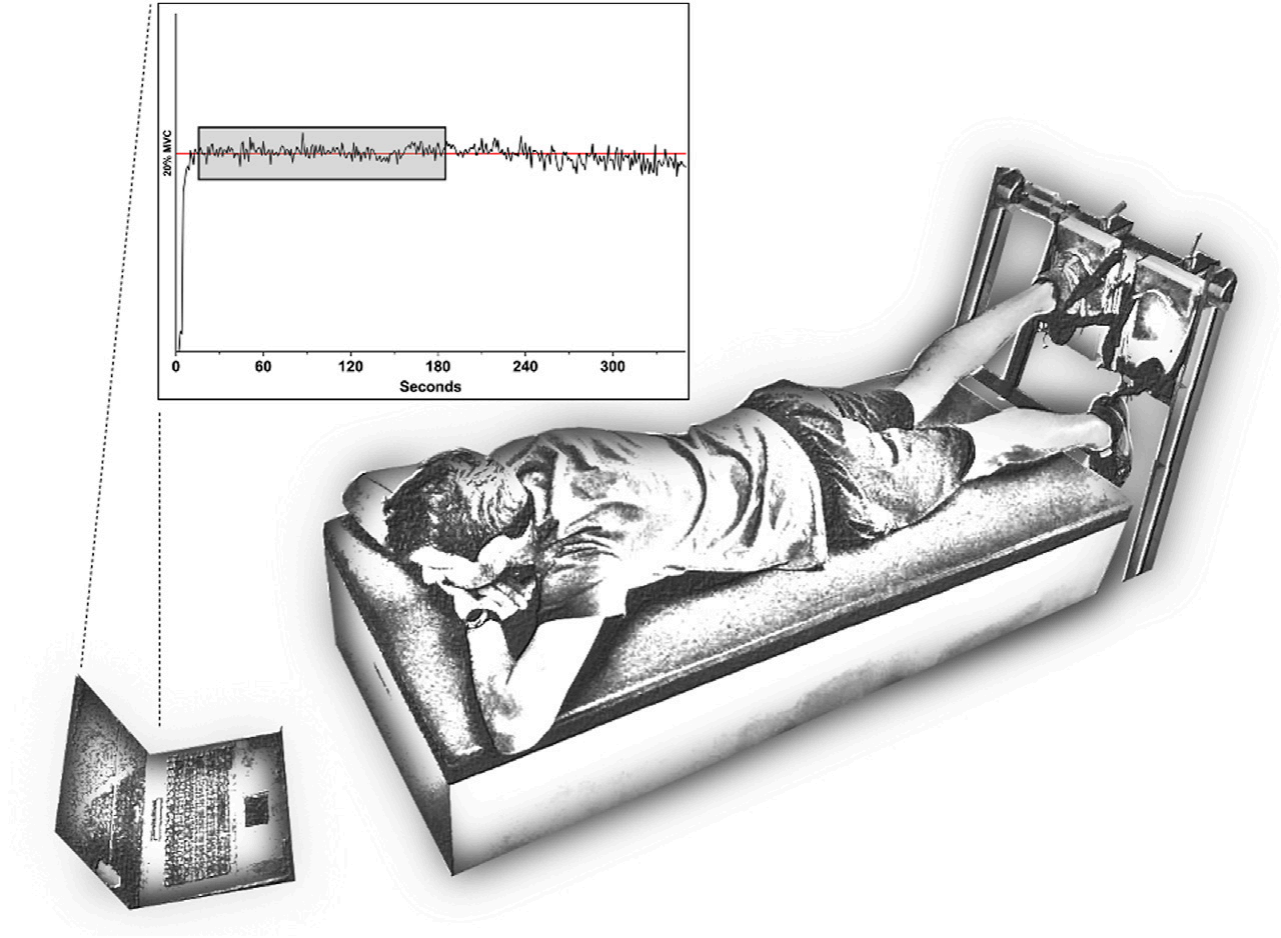
Experimental setup.

The participants completed one test session during the pre-season of 2017–2018. They were instructed not to perform strength or flexibility training for at least 72 h before the test sessions. Upon arrival to the laboratory, the participants were informed of the purpose of the study and the experimental protocol. For the experiment, they were positioned in the prone position, with the hips in a neutral position, and the tested knee flexed at 30° (0° = full extension). The foot of the tested limb was fixed in a foot holder, with the ankle at 90°, which contained a force transducer (Model STC, Vishay Precision, Malvern, PA) near the heel level to collect the linear force perpendicular to the leg orientation. The knee flexion force was measured at a sampling rate of 1,000 Hz using custom-made built equipment (Figure 1) (Mendes et al., 2020; Freitas et al., 2022). The signals were amplified (Model UA73.202, Sensor Techniques, Cowbridge, United Kingdom), digitally converted (USB-230 Series, Measurement Computing Corp., Norton, MA), recorded using the DAQami software (v4.1, Measurement Computing Corp., Norton, MA), and multiplied by the perpendicular distance between the force transducer center and the femoral lateral condyle, to calculate the knee flexion torque.

The participants were first familiarized with the experimental setup and then completed a warm-up protocol that comprised of around 20 submaximal knee flexion contractions at approximately 50% of perceived maximal intensity. The experiment first included two maximal voluntary isometric contraction (MVIC) knee flexion trials for both limbs with 1-min of rest in between. After 5 min rest, the participants performed a sustained submaximal isometric knee flexion at 20% of the MVIC level until failure. Failure was considered when the force produced decreased 5% from the required target contraction intensity for more than 5 s, or when the participant was unable to continue the task. Visual feedback of the force signals was provided and verbal encouragement was given to the participants through the course of the fatiguing task.

### Data analysis

If a participant was not able to maintain the force for a minimum of 5 min, the torque time series was excluded to avoid fatigue development that would be reflected in the torque data. This reduced the number of included participants to 5, 6. Furthermore, to ensure that the torque data represented a non-fatigue stage, only the first 183 s were used for further analysis. The initial 3 s were removed to avoid transient effects from rest-to-contraction.

The original and unfiltered torque time series sampled at 1,000 Hz and cropped to 180 s included a total of 180,000 data points. To determine the selection of the appropriate sampling frequency base on a power spectral analysis to establish which frequencies contain the majority of the information in the signal and on the operating time scale of the biological phenomenon in question, a power spectral analysis of each of the torque time series was completed. The 99.9% cut-off was recorded for each trial (Table 1).

**TABLE 1:** Power spectral density outcome.

	Frequency (Hz)
Group mean of the highest frequency	11.4
Group SD of the highest frequency	5.4
95% confidence interval	9.84–12.96
Maximal frequency across participants	21.9
Minimal frequency across participants	3.7

The time series were then downsampled to 750, 500, 250, 100, 50, and 25 Hz. From these, new time series were cropped with each sampling frequency to contain either 1) a fixed observation time of 180 s with a flexible total number of data points ranging from 180,000–4,500 or 2) a fixed number of data points of 4,500 for a flexible observation time ranging from 4.5–180 s. SaEn was calculated from all the cropped time series using the equation presented by Richman and Moorman (2000) with *m* = 2 and *r* = 0.2.

We then sought to determine the selection of appropriate input parameters (*m*, *r* and *N*) to ensure relative parameter consistency. Our power spectral analysis revealed a maximal frequency across all subjects of 21.9 Hz (Table 1). For any further analysis, we used a sampling frequency of 100 Hz following the recommendation by Stergiou (2004) of a sampling frequency five times greater than the highest frequency in the time series of interest. Using the time series sampled at 100 Hz with a 180 s duration, SaEn was calculated using combinations of *m* = 2 and 3 and *r* = 0.1, 0.15, 0.2, 0.25, 0.3, 0.35 and 0.4 times the standard deviation. Furthermore, using the time series sampled at 100 Hz, SaEn was calculated using *m* = 2 and *r* = 0.2 for six time series lengths of 500, 1,500, 3,000, 6,000, 9,000 and 18,000 data points.

Lastly, the selection of the appropriate observation time to capture sufficient information about the dynamics of the biological phenomenon was investigated. From the time series sampled at 100 Hz and with a duration of 180 s, consecutive windows of 5, 15, 30, 60 and 90 s were generated. SaEn was then calculated from the two windows using *m* = 2 and *r* = 0.2 for each window length.

## Statistics

To investigate the effect of different sampling frequencies on the SaEn, a one-way repeated measure ANOVA with sampling frequency as independent factor and SaEn as dependent variable was applied for both the fixed observation time condition and the fixed number of data points condition. In case of a significant effect of sampling frequency, a Holm-Sidak *post hoc* test was applied.

To investigate the effect of different input parameters *r* and *m* on the SaEn, a two-way repeated measure ANOVA with *r* and *m* as independent factors and SaEn as dependent variable was applied. In case of a significant effect of *r* or *m* or an *r-m* interaction, a Holm-Sidak *post hoc* test was applied. To investigate the effect of *N* on SaEn, a one-way repeated measure ANOVA with *N* as independent factor and SaEn as dependent variable was applied. In case of a significant effect of *N*, a Holm-Sidak *post hoc* test was applied.

Bland-Altman plot analysis was performed to quantify the agreement between the SaEn values from the first and second half of the five different observation times. The SaEn bias (i.e., mean within-subject difference), bias SD, bias 95% confidence intervals, limits of agreement and 95% confidence intervals of the upper and lower limit of agreement were calculated. Additionally, coefficient of repeatability was calculated as the 1.96 times the square root of the standard deviation of the within-subject difference. Low coefficient of repeatability indicates better repeatability of the SaEn from the first to the second half of the time series. Level of significance was set at 5%. All statistical calculations were performed in Sigmaplot (Systat Software, Inc. 2014, version 13.0, Germany).

## Results

**FIGURE 2:**
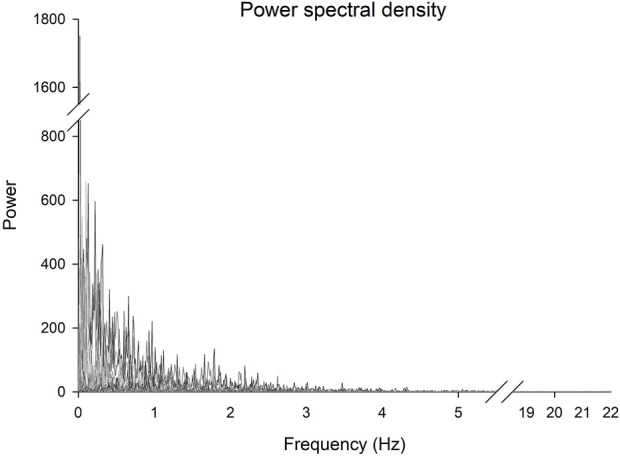
Power spectral density of the torque signals for each participant. Frequencies between 5.5 and 18.5 Hz and powers between 850 and 1,550 have been omitted.

The power spectral density analysis revealed a group mean of the highest frequency in the torque signals of 11.4 Hz and a maximal frequency across all participants of 21.9 Hz (Table 1; Figure 2).

**FIGURE 3:**
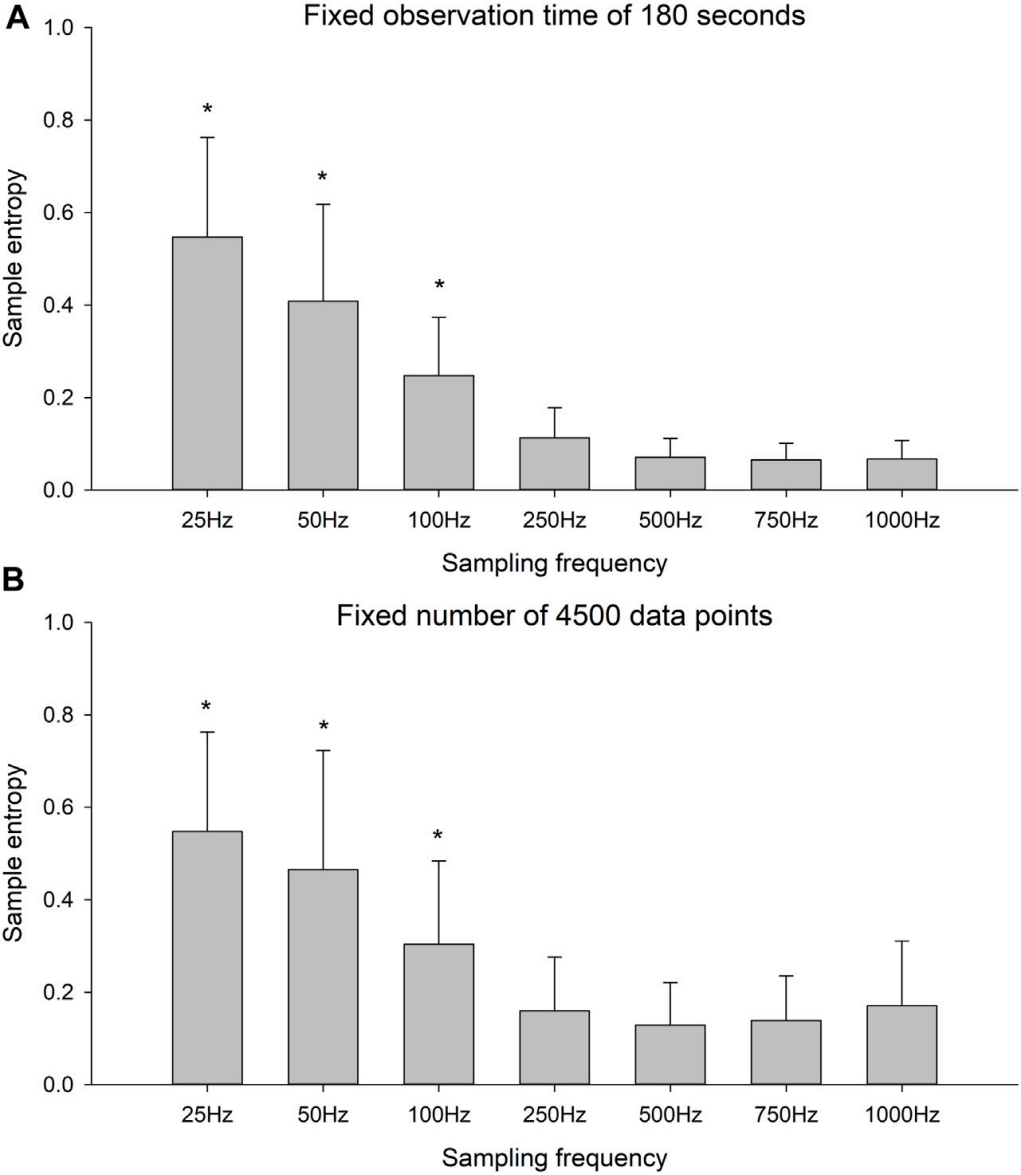
Sample entropy of the time series with six different sampling frequencies for **(A)** the fixed observation time of 180 s and **(B)** the fixed number of 4,500 data points. * indicates significant decrease in sample entropy with increment in sampling frequency.

There was a significant effect of sampling frequency for both the fixed observation time condition (Figure 3A, F-value = 227.9, *p* < 0.001) and the fixed number of data points condition (Figure 3B, F-value = 95.9, *p* < 0.001). The *post hoc* tests revealed that for both conditions, there were no differences in SaEn between the four highest sampling frequencies. With each decrement in sampling frequency from 250 Hz, the SaEn increased significantly (*p* < 0.001 for all decrement).

**FIGURE 4:**
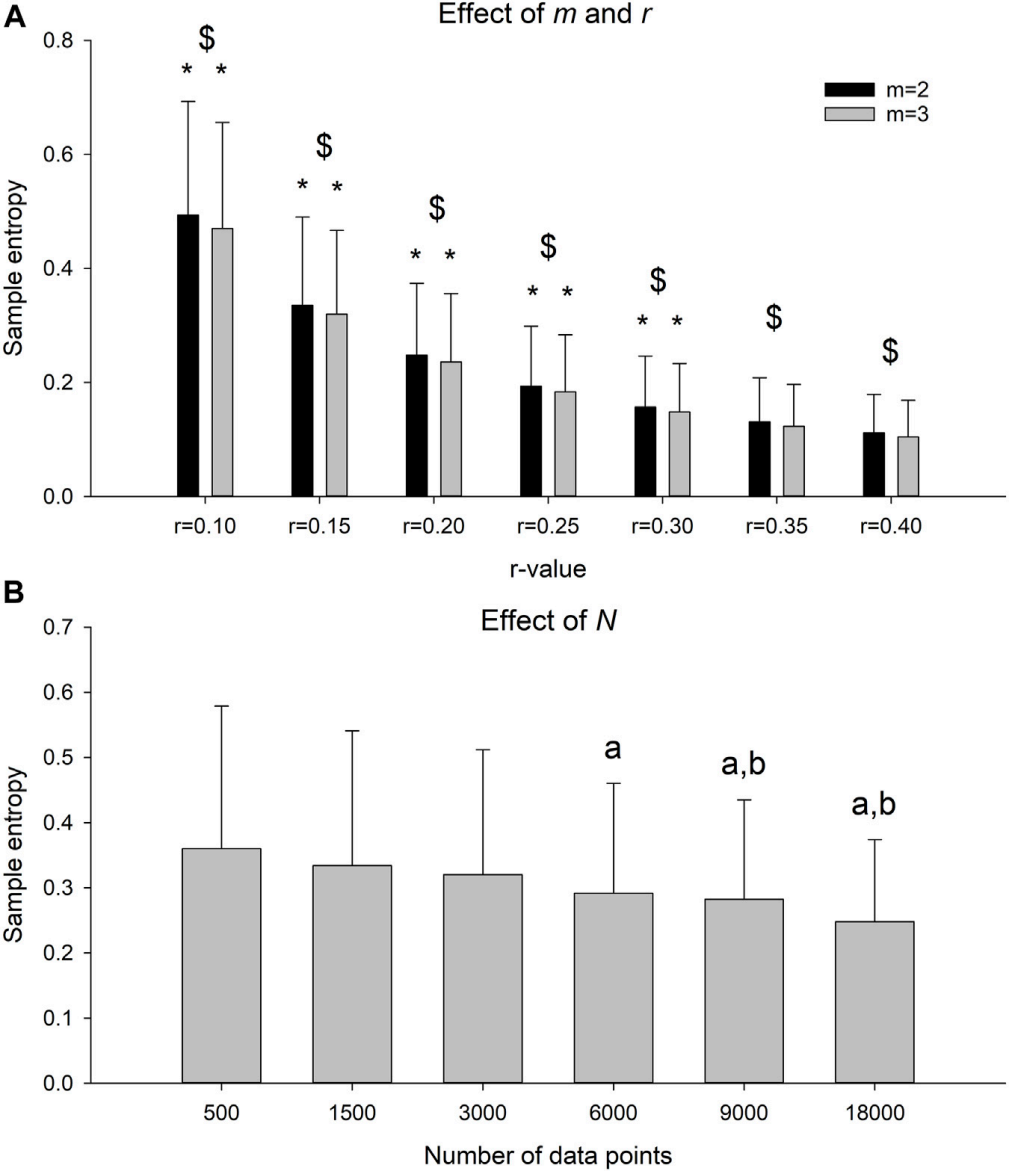
**(A)** Sample entropy of the time series sampled at 100 Hz for an observation time of 180 s with *m* = 2 or 3 and *r* = 0.10, 0.15, 0.20, 0.25, 0.30, 0.35 or 0.40. * indicates significant decrease in sample entropy with increase in *r* for a given *m*. $ indicates significant difference between *m* for a given *r*. **(B)** Sample entropy of the time series sampled at 100 Hz, with *m* = 2 and *r* = 0.2 and data lengths between 500 and 18,000 data points. A indicates significant different sample entropy from the time series with 500 data point and b indicates significant different sample entropy from the time series with 1,500 data point.

There was a significant *r-m* interaction on the SaEn (Figure 4A, F-value = 63.5, *p* < 0.001). For both *m* = 2 and *m* = 3, there was a significant decrease in SaEn with each increment in *r*-value (*p* < 0.022 for all comparisons). There was a significant effect of time series length on SaEn (Figure 4B, F-value = 11.4, *p* < 0.001). The SaEn of the time series with 500 data points was significantly higher compared to the 6,000, 9,000 and 18,000 data point time series (*p* < 0.001 for all comparisons). The SaEn of the time series with 1,500 data points was significantly higher compared to the 9,000 (*p* = 0.023) and 18,000 (*p* < 0.001) data point time series. No differences in SaEn were observed between time series with more than 1,500 data points.

**FIGURE 5:**
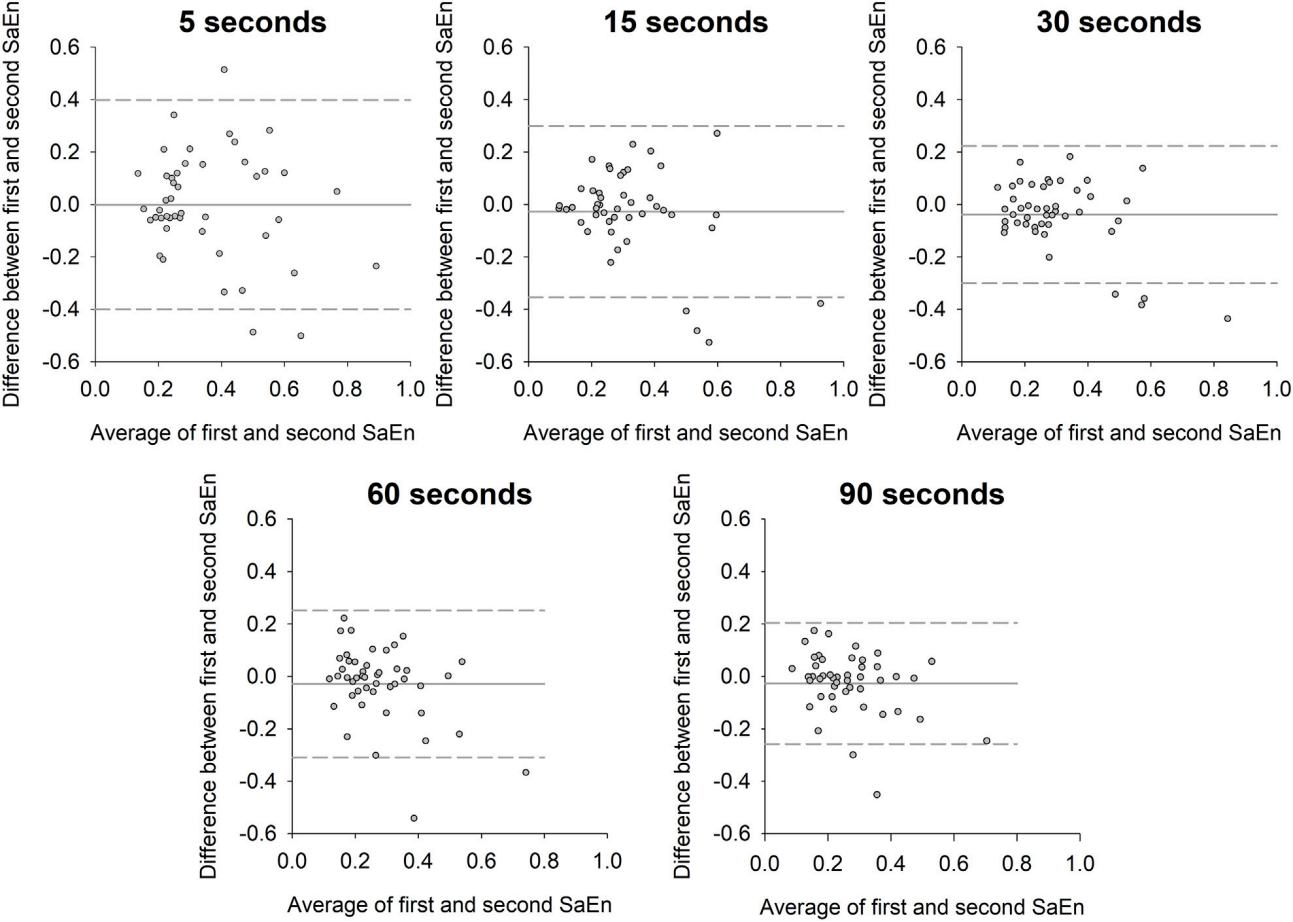
Bland-Altman plot of the difference in sample entropy between the first and second window of the time series against the average of the sample entropy from the two time series for the five different observation times. Solid horizontal line indicates sample entropy bias and dashed lines indicate upper and lower limits of agreement.

The Bland-Altman plot analysis revealed that the line of equality was within the 95% confidence intervals of the SaEn bias for all the five observation times (Figure 5; Table 2). The bias SD, the range of limits of agreement and the coefficient of repeatability decreased with increasing observation time.

**TABLE 2:** Bland-Altman analysis outcome for the comparisons of sample entropy calculated from the five different observation times. Sample entropy bias, bias SD, bias 95% confidence intervals, limits of agreement, 95% confidence intervals of the upper and lower limit of agreement and coefficient of repeatability.

t	5 s	15 s	30 s	60 s	90 s
Bias	−0.0007	**−**0.027	**−**0.039	**−**0.029	**−**0.027
SD	0.204	0.167	0.134	0.143	0.118
Bias 95% CI	**−**0.061–0.060	**−**0.077–0.022	**−**0.079–0.001	**−**0.071–0.014	**−**0.062–0.008
Limits of agreement	**−**0.400–0.399	**−**0.354–0.299	**−**0.300–0.227	**−**0.309–0.252	**−**0.258–0.204
Lower limit of agreement 95% CI	**−**0.505–-0.295	**−**0.440–-0.268	**−**0.369–-0.232	**−**0.383–-0.235	**−**0.318–-0.197
Upper limit of agreement 95% CI	0.294–0.503	0.214–0.385	0.154–0.291	0.178–0.325	0.144–0.265
Coefficient of repeatability	0.885	0.800	0.716	0.741	0.673

## Discussion

The purpose of the present study was to determine the appropriate methodological approach of the use of SaEn on isometric force or torque signals. We investigated 1) the effect of different sampling frequencies 2) the effect of different input parameters, and 3) the effect of different observations durations.

When recording biological signals, it is important to select an appropriate sampling frequency. First of all, the Nyquist theorem should be followed such that the sampling frequency is at least twice the size of the highest frequency presence in the signal of interest (Hamill et al., 1997). In the present study, we observed a maximal frequency in the torque time series of 21.9 Hz which would require a minimum sampling frequency of approximate 44 Hz. However, it has been recommended to use a sampling frequency of 4–6 times the highest frequency embedded in the signal to ensure an adequate representation of the time domain which led us to use 100 Hz for the later part of our analysis (Stergiou, 2004). While technological advancements have made it possible to record force and torque signals at higher sampling frequencies (e.g., above 1,000 Hz), this is not necessarily advisable. When recording signals from human movements such as force or torque, it is important to keep in mind that the nervous system does not have infinite resolution but modulation of muscle activity operates on a millisecond level (Lin et al., 1997; Sinkjær et al., 2000). Sampling with too high frequency when observing a phenomenon which evolves with low frequency oscillations could lead to the collection of redundant information (Yentes and Raffalt, 2021). The results of the present study suggest that torque data should be collected with sampling frequencies of a least 100 Hz, as lower frequencies for a fixed observation time of 180 s significantly decreases the SaEn. The results also suggest that torque data should not be collected at sampling frequencies beyond 250 Hz, as increasing the frequency further for a fixed number of data points of 4,500 did not change the SaEn. Thus, increasing the sampling frequency until 100 Hz provides greater details of the signal dynamics but increasing beyond 250 Hz does not add new information in terms of regularity suggesting that redundant information is collected at higher frequencies.

We have previously observed that the selection of input parameters affects continuous variables such as the torque collected in the present study data more than discrete variables (i.e., stride time intervals recorded from locomotion) (McCamley et al., 2018). This emphasizes the importance of investigating the effect of different input parameters on SaEn calculated from torque data. Furthermore, it is important to consider the interpretation of the vector length *m*. In a discrete time series, *m* = 2 or *m* = 3 represents two or three distinct observations of the phenomenon in question e.g., walking strides or heart beats. The recorded variable is then a time interval in seconds or a stride length in meters. For a continuous time series, the recorded variable (e.g., torque or force) is collected at a specific and constant time point given by the sampling frequency. In this case, the vector length represents a time interval related to the sampling frequency. In the present study, a sampling frequency of 100 Hz resulted in a vector length of *m* = 2 and *m* = 3 representing 20 and 30 milliseconds of the behavior of the phenomenon of interest. For a sampling frequency of 1,000 Hz, the corresponding vectors represented 1 and 2 milliseconds of behavior. With a maximal frequency of 21.9 Hz in the torque data, the minimum duration of oscillations within the signals was approximately 50 milliseconds. This means that the fastest oscillatory patterns within the signals will be detectable for 100 Hz and *m* = 3 because the longest duration of the compared vectors i.e., *m* = 3 and *m* = 3 + 1 will not exceed 50 milliseconds. However, it also means that increasing the sampling frequency will increase the number of repeated patterns detected within each oscillation decreasing the SaEn. This was observed until 250 Hz suggesting that increasing the sampling frequency further did not provide additional information regarding the regularity of the time series.

The SaEn decreased with increases in both *m* and *r* similar to what we have observed in previous studies when investigating different kinematic variables obtained from walking (Yentes et al., 2013; 2018; McCamley et al., 2018; Raffalt et al., 2019). The significant difference in SaEn between the 2 *m* values did not change direction when altering the *r*. This suggests relative parameter consistency when using the range of *r* in the present study. When designing studies including two or more experimental conditions or two or more groups, it is important to test the relative parameter consistency of between-group and between-condition differences. Thus, between-group and between-condition differences should be consistent across a range of input parameters and not change direction when input parameters are altered. Because the relative input parameter consistency for between-group and between-condition studies can be highly data specific, this should always be tested and reported (Yentes and Raffalt, 2021).

To fully capture the dynamics of any biological phenomenon, it is crucial that the observation time allows the phenomenon to unfold. To the best of our knowledge, there is no standardized way of determining the appropriate observation time. However, at least two parameters should be taken into account. First, the mean frequency of the signals in question is the inverse of the average duration of the oscillations of the signal. In the present study, the mean frequency was approximately 11 Hz which gives a mean oscillation duration of approximately 91 milliseconds. For the range of observation times from 5 to 90 s in the present study, this would result in between 55 and 989 oscillations, respectively. As the range of frequencies within the signal was relative large, it is unlikely that short observation time enables the capture of the behavior of all types of oscillations i.e., from high frequent to low frequent oscillations. Second, the motor control of movements relies on incorporation of sensory input relevant to the task in question. During the isometric contraction task, the muscle force is regulated based on visual input from the screen informing the participants of the target force and actual force and proprioceptive input from muscles and tendons around the knee joint (Tracy et al., 2007). These inputs are continuously accounted for in the generated muscle force (Nielsen, 2004). However, this process has an inherent delay of at least 40 milliseconds due to transmission time in afferent and efferent nerves, process time in higher order neural networks and spinal networks, electromechanical delay and rate of muscle force incline and decline (Nielsen, 2004; 2015; Debenham and Power, 2019; Schmid et al., 2019). This means that the neural modulations are reflected in the force oscillations below 25 Hz. Therefore, to fully capture the dynamics of the motor control requires sufficient observation time for these low frequency oscillations to unfold. Based on the results of the present study, at least 30 s of observation is required. Thus, the effect of data length for a fixed sampling frequency of 100 Hz on the SaEn revealed that using less than 3,000 data points (equal to an observation time of 30 s) would significantly affect the SaEn while using more than 3,000 data points would not. Furthermore, the results from the Bland-Altman plots indicated that the longer observation time, the better repeatability. Together this clearly suggests the observation time should be at least 30 s long and preferably longer.

In the present study, we only included a single relative low torque level. The use of higher torque levels would induce fatigue earlier during the isometric contraction and thereby reduce the observation time that is feasible for valid assessment of the torque dynamics. If at least 30 s of non-fatigued torque cannot by acquired at a given the torque level, we do not recommend calculating SaEn from the recorded time series. It should also be noted that results of the present study could be affected by the relative low torque level and the investigated muscle. Consequently, future studies should keep in mind that the results of the present study might not be generalizable across different torque levels or muscles. Furthermore, only torque signals recorded during isometric contractions were included in the present study and the generalization of the recommendations to other signals such as electromyography or electroencephalogram should be made with caution. We have previously provided guidelines for the application of entropy measures to kinematic signals from gait research (Yentes and Raffalt, 2021); however, future studies should explore the appropriate application of SaEn to other signals and during a wider range of movements. As discussed above, recording data with too high a sampling frequency can lead to the collection of redundant information. The present study did not filter the data prior to downsample or calculating sample entropy as this could remove biological information (Yentes and Raffalt, 2021). However, it should be noted that filtering can be appropriate when data is known to contain non-biological noise. As such it could be suggested to filter the data for higher frequencies (in this case above 5 Hz). To the best of our knowledge no formal test of data redundancy exists, and future work should aim at establishing this Based on the results of the present study, we can list three recommendations for future studies using SaEn to quantify regularity of low isometric force or torque signals: 1) the appropriate sampling frequency is between 100 and 250 Hz, 2) between-group and/or between-condition relative consistency of the input parameters *r*, *m*, and *N* should be tested and reported as SaEn changes with change in input parameter, and 3) the observation time should be at least 30 s to ensure the unfolding of the phenomenon in question.

## Data Availability

The datasets generated for this study are available on request to the corresponding author.
